# Double Tension Slide Technique as a Novel Repair for Distal Biceps Tendon Tear: A Biomechanical Evaluation

**DOI:** 10.7759/cureus.13895

**Published:** 2021-03-15

**Authors:** Kyle R Sochacki, Robert A Jack, Zachary T Lawson, David Dong, Andrew B Robbins, Michael R Moreno, Patrick McCulloch

**Affiliations:** 1 Orthopedics & Sports Medicine, Houston Methodist Hospital, Houston, USA; 2 Biomedical Engineering, Texas A&M University, College Station, USA; 3 Mechanical Engineering, Texas A&M University, College Station, USA

**Keywords:** distal biceps tendon repair, cortical button, biomechanics, failure strength

## Abstract

Background

A comparative biomechanical analysis of two distal biceps tendon repair techniques was performed: a single suture tension slide technique (TST) and two suture double tension slide (DTS) technique.

Methodology

Ten matched pairs of fresh frozen human cadaveric elbows (20 elbows) were randomly separated into two cohorts for distal biceps tendon repair. One cohort underwent the TST, and the other underwent the DTS technique. The tendon was preconditioned with cyclic loading from 0° to 90° at 0.5 Hz for 3,600 cycles with a 50 N load. The specimens were then loaded to failure at a rate of 1 mm/s. The difference in the load to failure between the groups was analyzed using the Student’s t test. The mode of failure was compared between groups using the chi-square test. All p-values were reported with significance set at p < 0.05.

Results

Overall, 77.8% of the included matched pairs demonstrated greater load to failure in the DTS group. The mean load to failure in the DTS group was 383.3 ± 149.3 N compared to 275.8 ± 98.1 N in the TST group (p = 0.13). The DTS specimens failed at the tendon (5/9), suture (3/9), and bone (1/9). The TST specimens failed at the tendon (4/9) and suture (5/9) only. There was no significant difference in failure type between groups (p = 0.76).

Conclusions

DTS demonstrates a similar to greater load to failure compared to TST with a trend towards statistical significance. The redundancy provided by the second suture has an inherent advantage without compromising the biomechanical testing.

## Introduction

Distal biceps tendon ruptures have increased in incidence and are typically secondary to an acute eccentric load on a degenerative tendon [1,2]. Non-operative treatment for these injuries leads to decreased forearm supination strength and to a lesser degree decreased elbow flexion strength [3-6]. Typically, operative intervention is recommended for patients who are young, active, and/or have an occupation in manual labor as repair may restore function to near normal levels [3,5-9].

Different surgical techniques exist for distal biceps repair which include the bone tunnel technique, suture anchor repair, interference screw, and cortical button [4,5,10-17]. Suspensory cortical button has been shown to have the highest load to failure between compared techniques [11-13,18-20]. The tension slide technique (TST) with an inference screw was then developed and exhibited greater load to failure and less gap formation compared to traditional fixation with cortical button [21].

The potential downside of utilizing an interference screw lies in the complication of fracture through the bone tunnel [22,23] As such, many have abandoned the use of interference screws and rely on single suture TST fixation with cortical button which has been shown to have good strength [21]. Those who employ this technique typically tie two strands of a single suture together which puts the entire repair at risk with rupture of a strand or loss of knot security. Concerns over the biomechanical strength led us to develop the double tension slide (DTS) technique using an additional suture with a cortical button. Prior biomechanical studies for distal biceps tendon repair techniques have not adequately analyzed the DTS technique.

The purpose of this study was to perform a comparative biomechanical analysis of two distal biceps tendon repair techniques: a single suture TST and two suture DTS technique. The authors hypothesize that there will be no difference between the two techniques.

## Materials and methods

Ten matched pairs of fresh frozen human cadaveric elbows (20 elbows) were obtained. Age, sex, radius diameter, and cadaveric weight and height were recorded. The ten matched pairs were then randomly separated into two cohorts. One cohort underwent a TST, as described by Sethi et al. [21]. The other cohort underwent DTS. The specimens were thawed at room temperature. A longitudinal incision was made along the medial border of brachioradialis, and the dissection was carried down to the distal biceps tendon insertion on the radial tuberosity. The distal biceps tendon was then sharply removed from the radial tuberosity.

The TST was performed using the technique described by Sethi et al. [21] without the use of an interference screw. A single No. 2 high tension non-absorbable composite suture (FiberLoop, Arthrex, Naples, FL, USA) with a straight needle was used to secure the distal 2.5 cm of the biceps tendon in a locking-loop fashion. Each limb of the suture was then passed through the biceps button (Arthrex, Naples, FL, USA) as described.

**Figure 1:**
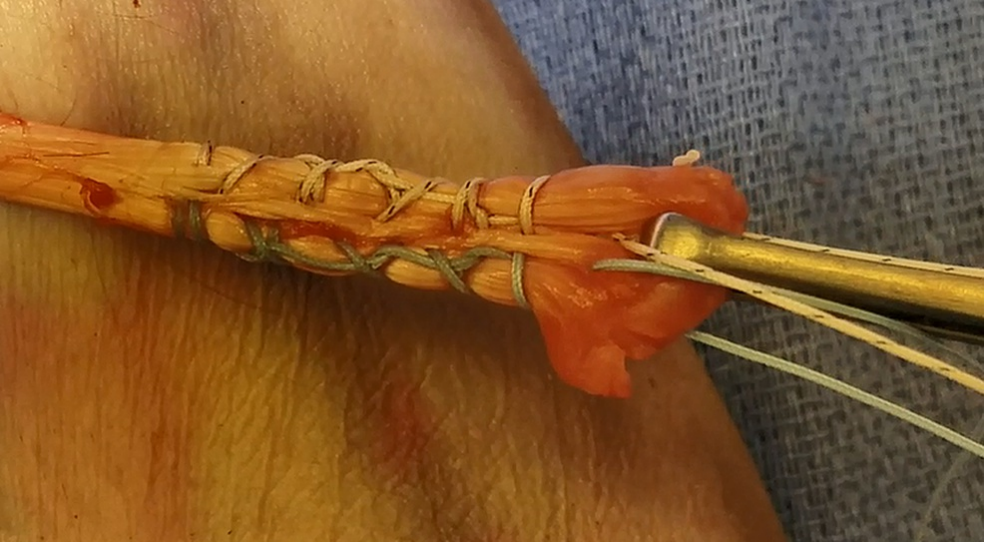
DTS technique with two sutures passed through the distal biceps tendon starting at the distal aspect of the tendon running proximally 2.5 cm in a locking fashion and then distally exiting 1 cm from the distal tendon. DTS, double tension side

The DTS technique was performed with two No. 2 high tension non-absorbable composite suture (FiberWire, Arthrex, Naples, FL, USA). We prefer to use a blue suture and tiger suture for ease of suture management. The first suture was passed through the distal biceps tendon starting at the medial and distal aspect of the tendon running proximally 2.5 cm in a locking fashion and then back distally exiting 1 cm from the distal tendon. This was then then repeated with the second suture starting at the lateral and distal aspect of the tendon (Figure 1). The two central strands of the sutures were then threaded through the cortical button (BicepsButton, Arthrex, Naples, FL, USA) in opposite directions. The central strand of the medial (tiger) suture was inserted through the medial hole and then back through the lateral hole. The central strand of the lateral (blue) suture was then inserted through the lateral hole and then back through the medial hole. Both strands that were passed through the biceps button were then facing toward the distal biceps tendon.

In both cohorts, a 3.2 mm guide pin (Arthrex, Naples, FL, USA) was drilled through the center of the radial tuberosity from anterior to posterior. The anterior cortex was then reamed with an 8.0 mm cannulated reamer (Arthrex, Naples, FL, USA). The guide pin was removed and a button inserted was used to pass the biceps button through the 3.2 mm hole in the tuberosity from anterior to posterior. The button was then released from the holder and the biceps button was “flipped.” The two limbs of suture passed through the button were then toggled to dock the biceps tendon into the bone socket. Once the biceps tendon was fully seated in the socket, the two limbs of the same suture were tied together for both the medial (tiger) and lateral (blue) sutures. Finally, the sutures that passed through the button (one tiger and one blue) were tied together to reinforce the construct.

The biomechanical testing was performed as previously described by Conroy et al. [24]. An effort was made to preserve as much of the soft tissue attachments as possible to preserve the normal physiological loading geometries. The elbow was mounted anatomically to a custom load frame. The proximal humerus was fixed in place by two bolts and positioned to allow full extension at the elbow. A threaded intramedullary screw was inserted into the distal radius. Weights were then added to the threaded rod until a 50 N force was obtained to simulate the expected contracted force of the biceps in the early postoperative period based on previous postsurgical studies [24].

The tendon was then attached to the custom testing system using a stainless-steel clamp and cycled from full extension to 90º flexion at a rate of 0.5 Hz for 3,600 cycles in a preconditioning fatigue protocol.

**Figure 2:**
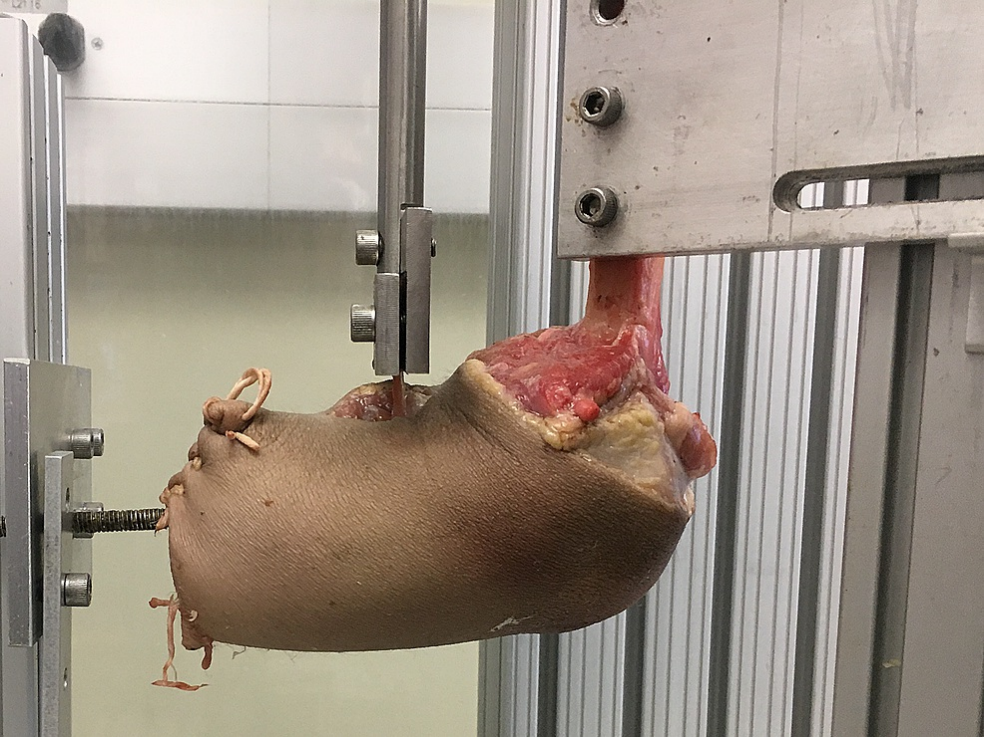
Custom mounting frame to load the distal biceps tendon repair to failure.

After cyclic loading was complete, the specimens were loaded to failure. The elbow was fixed at 90º flexion and the tendon loaded to failure at a rate of 1 mm/s (Figure 2). Failure was determined if the suture broke, bone fractured, or the tendon pulled free from suture, as defined by Mazzocca et al. [12]. The mechanism of failure and failure load was recorded. Specimens that sustained radial fracture at the interface of the threaded intramedullary screw prior to failure were excluded from analysis.

Following failure, the soft tissue was dissected to measure the diameter of the radius at the tuberosity. Individual group descriptive statistics were calculated using means ± standard deviation. The difference in the load to failure between the groups was analyzed using the Student’s t test. The mode of failure was compared between groups using the chi-square test. All p-values were reported with significance set at p < 0.05.

## Results

One matched specimen sustained radial shaft fracture at the bone-screw interface of the intramedullary screw prior to failure during cyclic loading, and both specimens of this matched pair were excluded from analysis. A total of nine matched (n = 18) pairs were included in the final analysis. All specimens were male (mean age of 48.9 ± 14.3 years, height 71.2 ± 2.6 in, weight 173.5 ± 44.4 lbs, and radius diameter 17.2 ± 0.9 mm).

**Figure 3:**
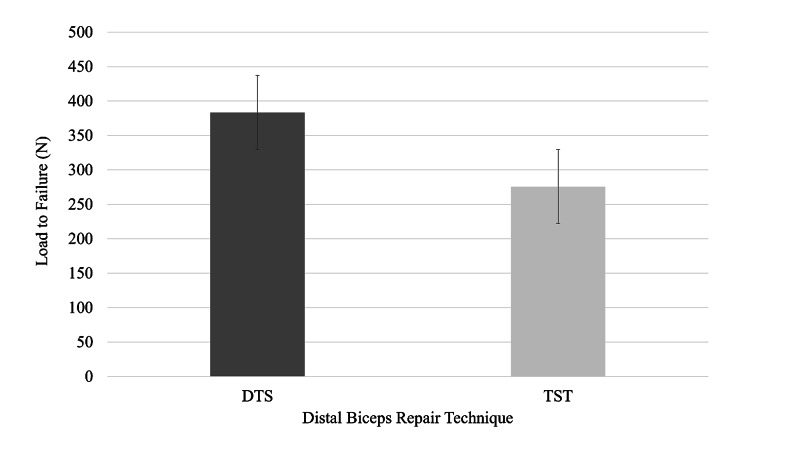
Comparison of the load to failure between the two techniques. TST, tension slide technique; DTS, double tension side; N, Newtons

None of the included specimens failed during preconditioning with cyclic loading. Overall, seven out of the nine (77.8%) included matched pairs demonstrated greater load to failure in the DTS group. The mean load to failure in the DTS group was 383.3 ± 149.3 N compared to 275.8 ± 98.1 N in the TST group (p = 0.13) (Figure 3). Overall, the DTS was 1.3 times (33.1%) stronger than the TST. In the specimens in which the DTS technique had a superior load to failure than the TST, the DTS technique was 1.6 times (68.3%) stronger.

The mode of failure for the DTS specimens occurred at the tendon (n = 5), suture (n = 3), and bone (n = 1). The specimens fixed with the TST failed at the tendon (n = 4) and suture (n = 5) only. There was no significant difference in failure type between groups (p = 0.76).

A post-hoc power analysis was performed for load to failure to determine the number of specimens needed to detect a significant difference. It demonstrated that for an alpha of 0.05 and power of 0.80, 22 specimens would be required in each group.

## Discussion

It was determined that the DTS technique and the TST had similar biomechanical strengths, with a trend towards the DTS technique having a superior load to failure. This study confirmed our hypothesis.

Several single and two incision techniques for distal biceps tendon repairs have been described with bone tunnels, suture anchors, interference screws, and cortical buttons [4,5,10-17]. Sethi et al. demonstrated that the single incision TST with interference screw had the strongest biomechanical properties [21]. However, reports of interference screw failure with increased risk of fracture through the bone tunnel led us to abandon its use and develop the DTS technique [12,22,23].

Intuitively, the addition of a second suture would likely increase the strength of the repair, but this had not been previously investigated. Furthermore, the redundancy of a second suture should provide a failsafe if either strand of the initial sutures was damaged by the needle, abrasion against the bone, or loss of knot security at the button.

The mean load to failure was similar between the DTS and TST groups (383. 3 ± 149.3 N vs. 275.8 ± 98.1 N, respectively). Although not statistically significant, there was a trend in favor of the DTS technique, with 77.8% of the matched pairs having superior strength compared to the TST. The loads to failure in the current study are similar to previous studies using suspensory cortical button fixation with the TST that ranged from 259 N to 440 N [12,20,21,25,26]. Due to the inherent differences of each biomechanical study (cadaver quality, testing machine, soft tissue envelope), it is difficult to perform inter-study comparisons. However, the fact that the average load to failure of the DTS technique was closer to the maximum range while that of the TST was closer to the minimum reported range provides further support in favor of the DTS technique for distal biceps tendon repairs.

Additionally, none of the included specimens failed during the preconditioning cyclic loading for 3,600 cycles. This was done with 50 N to simulate the expected contracted force of the biceps postoperatively while performing daily activities with the support of a brace [24]. This was similar to previous studies using the TST and cortical button fixation indicating that the DTS repair is strong enough to allow for range of motion in the early postoperative period [12,21].

The mechanism of repair failure did not differ between groups with similar numbers of suture breakage and tendon failure. This differs from the study by Sethi et al. in which the construct was more likely to fail at the tendon-suture interface compared to suture rupture [21]. Interestingly, both studies used the same suture. Additionally, by passing two sutures through the tendon, the present technique increases the cross-sectional area of the tendon in contact with suture to reduce the force transmitted through the individual passes of the suture and reduce failure at the tendon-suture interface. However, this topic requires further investigation.

There are limitations to this study. This study was limited by the small number of specimens and the associated low power. A post-hoc power analysis demonstrated that 22 matched pairs would be needed for an alpha of 0.05 and power of 0.80. This limitation is not unique to the current study, and it is commonly associated with biomechanical testing where obtaining an adequately powered sample size would be cost prohibitive. Nonetheless, the DTS load to failure was well within the range of other distal biceps tendon repairs using suspensory cortical fixation and is likely superior to the TST. We were unable to assess the bone mineral density (BMD) of the specimens for comparison between groups. However, due to the use of matched pairs, there is likely to be little variation in BMD between the left and right radius of the matched specimens. We also attempted to maintain as many soft tissue attachments as possible during the repair technique and biomechanical testing to preserve the normal physiological loading geometries. However, as with many biomechanical studies, the present study was unable to completely replicate the complex loading forces on the repaired tendon as seen in vivo with the combination of flexion, extension, pronation, and supination. Also, it is unclear what load to failure value is clinically relevant in the early postoperative period to prevent repair failure. As such, the stronger repair demonstrated by the DTS technique might not be clinically necessary assuming that the single suture and knot security are not compromised and should be investigated in future studies.

## Conclusions

The DTS technique demonstrates a similar to greater load to failure as the TST with a trend towards statistical significance. The redundancy provided by the second suture has an inherent advantage without compromising the biomechanical testing.
